# Understanding Adherence to Duloxetine in Psychiatric Practice: A Cross-Sectional Evaluation of Clinicians’ Experience

**DOI:** 10.3390/brainsci16020157

**Published:** 2026-01-29

**Authors:** Cielo García-Montero, Óscar Fraile-Martínez, Juan Pablo Chart-Pascual, Luis Gutiérrez-Rojas, Miguel Ángel Alvarez-Mon, Melchor Alvarez-Mon, Miguel Ángel Ortega

**Affiliations:** 1 Department of Medicine and Medical Specialities, Faculty of Medicine and Health Sciences, Network Biomedical Research Center for Liver and Digestive Diseases (CIBEREHD), University of Alcalá, 28801 Alcala de Henares, Spain; oscar.fraile@uah.es (Ó.F.-M.); maalvarezdemon@icloud.com (M.Á.A.-M.); miguelangel.ortega@uah.es (M.Á.O.); 2 Ramón y Cajal Institute of Sanitary Research (IRYCIS), 28034 Madrid, Spain; 3 Psychiatry Department, Osakidetza Basque Health Service, Araba University Hospital, 01009 Vitoria-Gasteiz, Spain; johnnychart@gmail.com; 4 Bioaraba Research Institute, 01009 Vitoria-Gasteiz, Spain; 5 University of the Basque Country UPV/EHU, 01006 Vitoria-Gasteiz, Spain; 6 Psychiatry Service, Hospital Universitario San Cecilio, 18016 Granada, Spain; gutierrezrojasl@hotmail.com; 7 Department of Psychiatry and CTS-549 Research Group, Institute of Neurosciences, University of Granada, 18071 Granada, Spain; 8 Centro de Investigación en Red de Salud Mental (CIBERSAM), 28029 Madrid, Spain; 9 Department of Psychiatry and Mental Health, University Hospital Infanta Leonor, 28031 Madrid, Spain; 10 Immune System Diseases-Rheumatology and Internal Medicine Service, University Hospital Príncipe de Asturias, 28805 Alcala de Henares, Spain

**Keywords:** duloxetine, adherence, major depressive disorder, anxiety, clinical psychiatry, patient satisfaction, antidepressants, serotonin and norepinephrine reuptake inhibitors (SNRIs)

## Abstract

**Objectives**: The present study aimed to explore psychiatrists’ perceptions of duloxetine in routine clinical practice, focusing on its efficacy, tolerability, and treatment adherence in major depressive disorder (MDD) and generalized anxiety disorder (GAD). **Methods**: A structured questionnaire was administered to 97 psychiatrists from different regions of Spain. The survey covered demographic and professional data, prescription frequency, perceived clinical efficacy, tolerability, dosing patterns, and factors influencing adherence. **Results**: Overall, duloxetine was perceived as an effective treatment for both MDD and GAD, particularly in patients with somatic symptoms or comorbid anxiety. Tolerability was also positively rated, with nausea and fatigue identified as the adverse effects most commonly associated with reduced adherence. In addition, patient education and close follow-up were identified as the most effective strategies to improve adherence, whereas digital tools were considered promising but underused. Compared with other antidepressants, duloxetine was viewed as having a favorable balance between efficacy and tolerability, with similar or slightly higher adherence rates. **Conclusions**: These findings reflect a positive clinical appraisal of duloxetine among psychiatrists, highlighting its role as a versatile therapeutic option for affective and anxiety disorders, within the context of routine clinical practice in Spain, provided that appropriate adherence-support strategies are implemented.

## 1. Introduction

Duloxetine is an antidepressant belonging to the class of serotonin and norepinephrine reuptake inhibitors (SNRIs). Through its dual mechanism of action, duloxetine increases the synaptic availability of both serotonin and norepinephrine in the central nervous system, contributing to the regulation of mood, anxiety, and pain-processing pathways [1]. This pharmacological profile differentiates duloxetine from selective serotonin reuptake inhibitors (SSRIs) and underlies its broad range of approved indications.

Due to this pharmacological profile, duloxetine has become a reference medication in the treatment of major depressive disorder (MDD) and generalized anxiety disorder (GAD), as well as in several chronic pain conditions such as diabetic peripheral neuropathy and fibromyalgia [2,3,4,5]. Because patients often present with coexisting affective symptoms and somatic or pain-related complaints, SNRIs such as duloxetine are frequently considered particularly suitable for these complex clinical profiles [6].

In clinical practice, duloxetine stands out for its therapeutic efficacy, tolerability, and versatility across diverse patient profiles, leading to its growing acceptance among psychiatric professionals [7,8]. However, the success of antidepressant treatment depends not only on pharmacological efficacy but also on treatment adherence, which remains a major challenge in the management of depressive and anxiety disorders [9,10]. Non-adherence is associated with poorer clinical outcomes, increased relapse rates, and higher healthcare costs [11,12,13].

Multiple factors influence adherence to antidepressant therapy, including adverse effects, perceived clinical improvement, dosing strategies, patient education, expectations, poor family support, the quality of the physician–patient relationship, healthcare system drawbacks and a lack of finances to access healthcare [13,14]. Importantly, clinicians’ prescribing attitudes and perceptions may directly shape how treatments are initiated, explained, and monitored, thereby indirectly affecting adherence and long-term outcomes [10]. Despite the availability of randomized controlled trials evaluating efficacy and safety, fewer studies have explored how psychiatrists perceive antidepressants in real-world settings, particularly concerning adherence-related factors.

Although collaborative care and patient-centered interventions have been developed to improve antidepressant adherence, evidence regarding their effectiveness remains mixed. Notably, some studies report improvements in depressive symptoms and quality of life without corresponding gains in long-term medication adherence [15]. This inconsistency underscores the complexity of adherence behavior and highlights the need for complementary real-world evidence focusing on clinicians’ prescribing attitudes and monitoring practices.

Recent evidence suggests that precision prescribing strategies, such as pharmacogenetic-guided (PGx) antidepressant treatment, may enhance functional outcomes and quality of life in patients with anxiety and affective disorders compared with standard treatment approaches. Although such strategies hold promise, available trials are limited in number and sample size, with variable methodological quality, highlighting the need for complementary real-world data on treatment selection, adherence, and clinical decision-making. Understanding psychiatrists’ perceptions of duloxetine—including its efficacy, tolerability, and adherence-related considerations—can provide valuable insights to inform clinical practice and support the rational use of antidepressants in diverse patient populations [16].

In this context, understanding psychiatrists’ perceptions of duloxetine is essential to clarify its current role in clinical practice. The present study aims to explore psychiatrists’ experiences, attitudes, and evaluations regarding the efficacy, tolerability, and adherence associated with duloxetine in patients with MDD and GAD. Additionally, it seeks to identify key factors influencing its selection over other antidepressants. By capturing real-world clinical perspectives, the findings provide valuable insights to optimize prescribing practices, improve adherence, and support the rational use of duloxetine in affective and anxiety disorders.

Despite prior survey-based research on antidepressant prescribing attitudes, there is a lack of recent national data specifically examining psychiatrists’ perceptions of duloxetine-related adherence in routine practice in Spain [17,18]. Moreover, no studies have systematically explored clinicians’ views on adherence-support strategies, including dosing approaches, patient education, and digital tools, in the context of duloxetine treatment. Accordingly, this study was designed to address the following research questions:

(1) How do psychiatrists perceive adherence to duloxetine in routine clinical practice, and how is it perceived relative to other antidepressants?
(2) Which clinical strategies (e.g., dosing, titration, patient education, follow-up practices) are most frequently used by psychiatrists to support adherence to duloxetine?
(3) What factors do psychiatrists identify as the main barriers and facilitators of treatment adherence in patients treated with duloxetine?

## 2. Materials and Methods

### 2.1. Study Design

A cross-sectional observational study was conducted to analyze psychiatrists’ perceptions of the factors influencing adherence to duloxetine treatment in patients with depressive and/or anxiety disorders. The study was based on the clinical experience and professional judgment of the participants, aiming to capture diverse real-world clinical perspectives.

### 2.2. Participants and Ethical Concerns

The target population consisted of licensed medical doctors specialized in psychiatry who were actively involved in the clinical management of patients with depressive and/or anxiety disorders. Eligible participants were aged between 25 and 65 years and had varying levels of professional experience.

Participants were recruited using a non-probabilistic, voluntary sampling strategy through an open call disseminated via national medical associations and professional communication platforms commonly used by psychiatrists in Spain. This approach was chosen to facilitate broad geographic reach and participation across different healthcare settings, including public and private practice.

Because the survey was distributed through open professional channels, the exact number of psychiatrists who received the invitation could not be determined, and a formal response rate could not be calculated. Consequently, the sample may be subject to self-selection bias. A total of 97 complete and valid responses were collected and included in the analysis. Detailed demographic and professional characteristics of respondents are reported to allow contextualization of the sample and assessment of its diversity.

The study was conducted in accordance with ethical standards, though formal Institutional Review Board approval was deemed unnecessary because participants were practicing professionals contributing expert judgment rather than patients. All data were collected via a voluntary, anonymized questionnaire that excluded personal health information or identifiable data. Consistent with the non-interventional nature of the study, verbal informed consent was obtained from all participants prior to their inclusion, with anonymity and voluntary participation ensured throughout the process.

### 2.3. Data Collection

Data were collected using a structured, self-administered questionnaire consisting of 25 multiple-choice items designed specifically for this exploratory study. The questionnaire aimed to capture psychiatrists’ clinical perceptions and routine practice experiences regarding duloxetine use and treatment adherence, rather than to function as a psychometric measurement instrument.

Questionnaire content was informed by a targeted review of the literature on antidepressant adherence and prescribing practices, as well as by themes explored in prior national clinician survey studies conducted by the authors’ group. To ensure content validity, clinical relevance, and clarity, the initial questionnaire draft was reviewed by an expert panel composed of board-certified psychiatrists with extensive experience in the treatment of mood and anxiety disorders and routine use of antidepressant pharmacotherapy. Panel members evaluated the questionnaire for relevance of domains, clarity of wording, and alignment with real-world clinical practice. Based on their feedback, minor revisions were made to improve item wording and reduce ambiguity. No items were removed, and no additional items were added after this review.

Given the descriptive and exploratory nature of the study, formal psychometric validation procedures (e.g., factor analysis or internal consistency testing) were not performed.

This questionnaire was custom-designed to gather qualitative insights from clinicians; it is not a psychometrically validated assessment tool.

The questionnaire was divided into the following sections:

(i) **Demographic and Professional Data** (Questions 1–5), including sex, age, province, year of residency completion, and current workplace;
(ii) **Prescription Frequency and Clinical Profile** (Question 6), addressing how often duloxetine is prescribed and in which patient profiles;
(iii) **Clinical Efficacy and Perceived Usefulness** (Questions 7–9), evaluating perceived efficacy in major depressive disorder and generalized anxiety disorder, as well as patient subgroups in which duloxetine is considered most effective;
(iv) **Tolerability and Adverse Effects** (Questions 10–12), focusing on adverse effects limiting adherence, reasons for treatment discontinuation, and the frequency of adverse events leading to dropout;
(v) **Dosage and Administration Factors** (Questions 13–15), including preferred dosing regimen, time of day for administration, and average time until perceived clinical improvement;
(vi) **Adherence Factors and Improvement Strategies** (Questions 16–20), assessing psychiatrists’ views on factors influencing adherence and the perceived effectiveness of strategies such as patient education, monitoring, and digital tools; and
(vii) **Psychiatrists’ Perception of Patient Adherence** (Questions 21–25), covering observed adherence rates, frequency and causes of treatment discontinuation, comparisons with other antidepressants, and the impact of structured follow-up.

The full questionnaire is provided as Supplementary Materials S1, in its original Spanish version.

All responses were collected anonymously, and no identifying information was recorded.

### 2.4. Data Analysis

Data were analyzed using descriptive statistics. Absolute frequencies and relative percentages were calculated for all questionnaire items. For key proportions, 95% confidence intervals (CIs) were computed to improve interpretability.

In addition to descriptive analyses, exploratory subgroup analyses were performed to examine whether psychiatrists’ perceptions differed according to selected demographic and professional characteristics, including age, sex, years of clinical experience, and region of practice. Years of professional experience were calculated up to 2025 based on the reported year of residency completion and categorized into four groups: <5 years, 5–10 years, 10–20 years, and >20 years.

Exploratory inferential analyses were conducted using chi-square tests or Fisher’s exact tests, as appropriate, to examine associations between psychiatrist characteristics and selected perception-based outcomes. For key proportions, 95% confidence intervals were calculated. Given the exploratory nature of the study and limited sample size, these analyses were considered hypothesis-generating. No correction for multiple comparisons was applied, and *p*-values should be interpreted descriptively rather than as confirmatory evidence.

Data visualization and graphical representations were generated using Python (version 3.x) employing the matplotlib library.

## 3. Results

### 3.1. Demographic and Professional Characteristics

A total of 97 psychiatrists participated in the survey. The sample included both men and women, with ages ranging from 30 to 65 years. Women constituted the majority of respondents (58.8%, *n* = 57), while men accounted for 41.2% (*n* = 40). Most participants were relatively early- to mid-career professionals, with over half of the sample aged between 30 and 45 years (55.7%), while the remaining respondents were distributed across the 46–55 and 56–65 age groups, reflecting a broad range of clinical experience. Years of professional experience showed a wide distribution, ranging from early-career psychiatrists to highly experienced clinicians, allowing for an exploration of perceptions across different career stages.

Regarding professional practice setting, the vast majority of psychiatrists reported working primarily in outpatient or ambulatory care settings (89.7%, *n* = 87). Smaller proportions of respondents practiced in acute inpatient units (6.2%, *n* = 6), day hospitals (3.1%, *n* = 3), or subacute inpatient units (1.0%, *n* = 1). This distribution reflects the predominance of outpatient psychiatric care in routine clinical practice and provides relevant context for the interpretation of perceptions related to antidepressant prescribing, tolerability, and treatment adherence in real-world settings.

Participants were recruited from multiple Autonomous Communities across Spain. The most represented regions were Andalusia (*n* = 20, 20.6%), Galicia (*n* = 14, 14.4%), Catalonia (*n* = 12, 12.4%), the Valencian Community (*n* = 10, 10.3%), and the Community of Madrid (*n* = 10, 10.3%). The remaining participants were distributed across other regions, each contributing smaller numbers, reflecting broad national coverage.

### 3.2. Frequency and Profile of Use

Regarding prescribing patterns, 70% of respondents reported prescribing duloxetine in 20–50% of their patients with depression or anxiety, while 28% do so frequently (in more than 50% of cases). Only 2% rarely prescribe it, and none indicated not using it at all.

### 3.3. Efficacy and Clinical Perception

In terms of perceived efficacy, 67% rated duloxetine as highly effective for the treatment of major depressive disorder (MDD), while 33% considered its efficacy moderate.

For generalized anxiety disorder (GAD), 76% of psychiatrists rated duloxetine as fairly effective, 16% as very effective, and 7% as slightly effective. When asked about patient profiles, 75% of participants reported greater efficacy in patients with depression accompanied by somatic or pain symptoms, 15% in those with comorbid anxiety, 4% in depression without anxiety, and 5% perceived no significant differences among profiles.

### 3.4. Tolerability and Adverse Effects

According to the respondents, the most common adverse effects perceived to limit adherence were nausea and gastrointestinal discomfort (53%), sexual dysfunction (24%), and dizziness or fatigue (18%). Adverse events leading to discontinuation were described as rare by 52% of psychiatrists, and occasional by 42%. Psychiatrists identified intolerable side effects (38%) and insufficient education about the treatment (41%), while 21% cited lack of efficacy.

### 3.5. Dosage and Administration Factors

To optimize adherence, the preferred initial dosing regimen was 30 mg/day with subsequent titration (78%), followed by 60 mg/day from the start (10%). Respondents associated morning administration with better adherence (57%) compared to evening intake (1%). Over half of respondents (56%) reported observing clinical improvement between 2 and 4 weeks, and 37% between 4 and 8 weeks after initiation.

### 3.6. Adherence Factors and Improvement Strategies

The main factors influencing adherence were perceived treatment efficacy (52%) and patient education about the illness and medication (29%). When asked to compare duloxetine with other antidepressants commonly used in their routine practice, respondents primarily referred to comparisons with other SNRIs—most frequently venlafaxine—as well as with first-line SSRIs based on their overall clinical experience rather than formal head-to-head switching protocols.

When comparing treatments, 41% of respondents perceived better adherence with duloxetine than with other SNRIs like venlafaxine (n = 40; 95% CI: 31.9–51.2%). A total of 40% reported it was similar and only 1% reported it as worse. These comparisons reflect psychiatrists’ global clinical impressions derived from routine prescribing and follow-up, rather than objective adherence metrics or structured comparative trials.

The most useful strategies to enhance adherence were providing detailed education on benefits and side effects (47%) and initiating treatment with low doses followed by gradual adjustment (43%).

### 3.7. Psychiatrists’ Perceptions of Patient Adherence

According to 90% of respondents, fewer than 20% of their patients discontinue duloxetine within the first three months. Furthermore, 76% indicated that discontinuation due to lack of response is uncommon. Overall, 54% perceive that patients find duloxetine more effective and better tolerated than other antidepressants, while 38% notice no significant differences. In terms of clinical decision-making, 42% recommend duloxetine in patients with previous adherence difficulties, and 56% tailor their recommendation based on patient profile. A majority (87%) agreed (“yes” or “probably yes”) that adherence would improve with a more structured follow-up, including phone calls or reminders.

Finally, 60% reported observing high patient satisfaction after more than six months of treatment, and 41% recommend duloxetine as a first-line option for depression with comorbid anxiety, while 43% do so depending on clinical presentation.

### 3.8. Exploratory Subgroup Analyses and Summary of the Most Relevant Psychiatrists’ Perceptions

**Figure 1:**
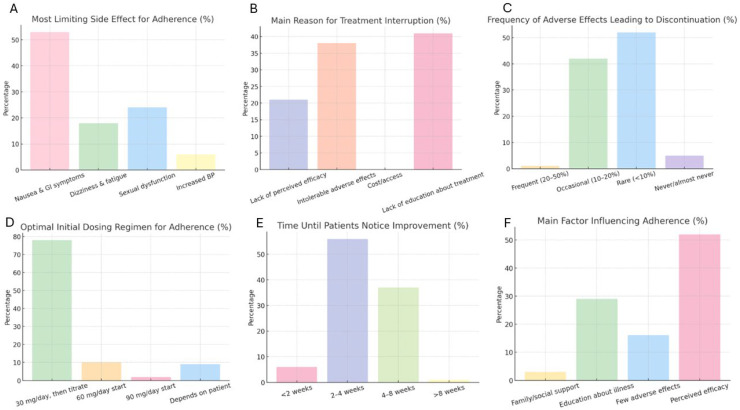
Summary of key survey findings regarding duloxetine adherence and prescribing practices (N = 97 psychiatrists). (**A**) Most limiting adverse effects for treatment adherence, with gastrointestinal symptoms (nausea and gastrointestinal discomfort) identified as the primary barrier. (**B**) Main reasons for treatment interruption, highlighting lack of education about treatment and intolerable adverse effects as the most frequently reported causes. (**C**) Frequency of adverse effects leading to treatment discontinuation, as perceived by psychiatrists, showing that discontinuation due to adverse events was most commonly reported as rare (<10%). (**D**) Preferred initial dosing regimen to optimize adherence, demonstrating a strong preference for starting at 30 mg/day followed by gradual titration. (**E**) Time until patients are perceived to notice clinical improvement, most frequently between 2 and 4 weeks after treatment initiation. (**F**) Main factors influencing adherence, with perceived treatment efficacy and patient education identified as the most important determinants. Values represent the percentage of respondents selecting each option. Percentages are calculated within the total sample. The figure provides a quantitative visual summary of clinicians’ perceptions and does not represent objective adherence or comparative effectiveness outcomes.

Some of the most relevant observations obtained from this questionnaire are summarized in Figure 1.

Exploratory subgroup analyses were conducted to examine whether psychiatrists’ perceptions of duloxetine varied according to sex, age group, practice setting, and years of professional experience (Table 1). Given the exploratory nature of the study and small sample sizes in some categories, these analyses are hypothesis-generating.

**Table 1:** Exploratory descriptive subgroup analysis of psychiatrists’ perceptions of duloxetine by demographic and professional characteristics (sex, age group, and practice setting).

Subgroup	n	Perceived High Efficacy n (%); 95% CI	*p* Value	Preferred 30 mg Start n (%); 95% CI	*p* Value	Better Adherence vs. Other SNRIs n (%); 95% CI	*p* Value
**Sex**							
Men	40	33 (82.5%); [68.1–91.3%]	**0.012**	31 (77.5%); [61.5–88.2%]	1	19 (47.5%); [32.0–63.4%]	0.401
Women	57	32 (56.1%); [42.1–69.3%]		45 (78.9%); [66.0–88.0%]		21 (36.8%); [25.1–50.2%]	
**Age group (years)**							
25–35	24	14 (58.3%); [37.2–77.3%]	0.18	19 (79.2%); [57.8–92.9%]	0.91	10 (41.7%); [22.1–63.4%]	0.22
35–45	37	20 (54.1%); [37.0–70.4%]		30 (81.1%); [22.1–63.4%]		7 (18.9%); [8.0–35.2%]	
45–55	22	17 (77.3%); [54.6–92.2%]		18 (81.8%); [59.7–94.8%]		11 (50.0%); [28.2–71.8%]	
55–65	14	11 (78.6%); [49.2–95.3%]		10 (71.4%); [41.9–91.6%]		7 (50.0%); [23.0–77.0%]	
**Practice setting**							
Outpatient	87	56 (64.4%); [53.0–74.7%]	0.17	70 (80.5%); [70.0–88.7%]	0.19	36 (41.4%); [31.0–52.3%]	0.94
Inpatient/hospital-based	10	9 (90%); [55.5–99.7%]		6 (60%); [26.2–87.8%]		4 (40%); [12.2–73.8%]	
Years of professional experience (until 2025) * (n = 93)							
<5 years	17	10 (58.8%); [32.9–81.6%]	0.09	16 (94.1%); [71.3–99.9%]	0.74	9 (52.9%); [27.8–77.0%]	0.11
6–10 years	21	16 (76.2%); [52.8–91.8%]		18 (85.7%); [63.7–97.0%]		5 (23.8%); [8.2–47.2%]	
11–20 years	34	20 (58.8%); [40.7–75.4%]		24 (70.6%); [52.5–85.0%]		14 (41.2%); [25.5–58.0%]	
>20 years	21	17 (81.0%); [58.1–94.6%]		14 (66.7%); [43.0–85.4%]		11 (52.4%); [29.8–74.3%]	

Values are presented as n (%), calculated within each subgroup. *p*-values are derived from χ^2^ tests or Fisher’s exact tests, as appropriate based on cell size. All analyses are exploratory and hypothesis-generating; *p*-values are reported for descriptive purposes only and were not adjusted for multiple comparisons. Confidence intervals are provided in Table 2. Results should be interpreted cautiously given the exploratory design and small sample sizes in some subgroups. Practice settings were collapsed to avoid sparse cells. Four responses had missing data for years of professional experience and were excluded from those analyses. Across the entire sample, 41.2% of psychiatrists (n = 40; 95% CI: 31.9–51.2%) perceived better adherence with duloxetine compared with other SNRIs. All subgroup comparisons should be interpreted cautiously due to small sample sizes and exploratory design. * Four responses had missing or non-valid year data and were excluded from the calculation of “years of professional experience”. Bold highlights the significance of the *p*-value.

**Table 2:** Key Survey Outcomes with 95% Confidence Intervals (CIs), highlighting exploratory patterns across key subgroups.

Outcome	Overall n (%)	95% CI	Notes/Subgroup Insights
Psychiatrists perceiving duloxetine as highly effective for MDD	65 (67.0%)	57.1–75.8	Consistent with previous surveys [17,18]; perceived efficacy tended to increase with years of professional experience
Psychiatrists perceiving duloxetine as highly effective for GAD	74 (76.0%)	66.4–83.6	Similar trends observed in patients with comorbid pain or somatic symptoms [2,3,4,17,19]
Preference for 30 mg/day starting dose	76 (79%)	68.8–85.7	Aligns with evidence that gradual titration may reduce nausea and early discontinuation [14,18,20]
Better adherence vs. other SNRIs	40 (41%)	31.9–51.2	Slightly lower than previous 80-psychiatrist survey (69%) [18], though directionally consistent
Adherence drivers: perceived efficacy	50 (52%)	42.0–61.7	Reinforces perceived effectiveness as a key determinant of treatment continuity [21]
Adherence drivers: patient education	28 (29%)	20.9–38.4	Supports the role of structured education and follow-up
Male psychiatrists reporting high perceived efficacy	33 (82.5%)	68.1–91.3	Exploratory analyses suggested higher perceived efficacy among male psychiatrists; findings should be interpreted cautiously. See Table 1 for female psychiatrists and other subgroup analyses.

**Sex:** Male psychiatrists reported perceived high efficacy of duloxetine more frequently than female psychiatrists (82.5%, n = 33; vs. 56.1%, n = 32). An exploratory analysis suggested a potential signal of higher perceived efficacy among male psychiatrists compared with female psychiatrists. The proportion of psychiatrists preferring a starting dose of 30 mg/day was similar between men and women (77.5%, n = 31; vs. 78.9%, n = 45). Perceived better adherence with duloxetine compared with other SNRIs was reported by 47.5% of men (n = 19) and 36.8% of women (n = 21).

**Age Group:** High perceived efficacy of duloxetine increased with age: 58.3% (n = 14) among 25–35 years, 54.1% (n = 20) among 35–45 years, 77.3% (n = 17) among 45–55 years, and 78.6% (n = 11) among 55–65 years. Preference for starting at 30 mg/day was consistently high across all age groups (71.4–81.8%), while perceived better adherence with duloxetine compared with other SNRIs was more frequently reported in older age groups (50% in 45–55 and 55–65 years). Due to small cell sizes, Fisher’s exact tests were applied; no robust exploratory patterns supporting age-related differences emerged, and inferential results are presented descriptively without correction for multiple comparisons.

**Practice Setting:** Outpatient psychiatrists reported high perceived efficacy in 64.4% of cases (n = 56), compared with 90.0% (n = 9) among inpatient/hospital-based psychiatrists. Preference for a 30 mg/day starting dose was higher in outpatients (80.5%, n = 70) than in hospital-based clinicians (60.0%, n = 6). Perceived better adherence was similar between groups (41.4% vs. 40.0%). Fisher’s exact tests showed no statistically significant differences (*p* > 0.05).

**Years of Professional Experience:** Perceived high efficacy increased with experience, from 58.8% (n = 10) in those with <5 years, to 81.0% (n = 17) in those with >20 years. Preference for initiating at 30 mg/day remained high across all experience groups (66.7–94.1%), while better adherence with duloxetine vs. other SNRIs was reported more frequently by psychiatrists with >20 years of experience (52.4%, n = 11) than by those with 6–10 years (23.8%, n = 5). Given the exploratory nature of the analyses and small subgroup sizes, inferential results did not indicate consistent exploratory signals across experience categories, and all findings are reported descriptively.

## 4. Discussion

The present survey provides a detailed, real-world overview of psychiatrists’ perceptions regarding the use of duloxetine in routine clinical practice, highlighting its perceived efficacy, tolerability, and impact on adherence in patients with MDD and GAD. Overall, our results align with existing clinical evidence and prior surveys conducted in Spain, while adding new quantitative insights through subgroup analyses and 95% confidence interval reporting.

### 4.1. Perceived Efficacy and Patient Profiles

Consistent with prior research, the majority of psychiatrists in the current survey rated duloxetine as highly effective for MDD (67%) and GAD (76%), with a particular preference for patients presenting depression accompanied by somatic or pain symptoms (75%). This is in line with our previous national survey of 163 psychiatrists, in which 70% similarly identified duloxetine as particularly effective in patients with somatic comorbidities [2,3,4,17,19]. In the smaller survey of 80 psychiatrists focused on clinical attitudes, 69% also reported superior efficacy in these patient profiles. These findings reflect a stable and consistent perception among Spanish psychiatrists regarding duloxetine’s effectiveness, particularly in patients where pain and mood symptoms co-occur.

Stratified analyses in the current study revealed an increase in perceived efficacy with higher levels of professional experience, ranging from 58.8% among psychiatrists with <5 years of practice to 81.0% among those with >20 years, underscoring potential influence of clinical exposure on treatment judgments—a nuance not captured in prior surveys [17,18].

### 4.2. Tolerability and Adverse Effects

Gastrointestinal adverse events, especially nausea, were reported as the main factor limiting adherence by 53% of respondents, followed by sexual dysfunction (24%) and fatigue/dizziness (18%). These observations mirror previous findings in both our surveys, where gastrointestinal symptoms were consistently the most cited barrier to adherence. The similarity in reported tolerability profiles reinforces the reliability of clinician-perceived adverse effects and aligns with controlled clinical trial data, which indicate gastrointestinal complaints as the leading cause of early discontinuation [14,18,20].

### 4.3. Adherence and Improvement Strategies

Adherence emerged as a key focus in this survey. Perceived treatment efficacy (52%) and patient education (29%) were identified as the main drivers of adherence, reflecting patterns observed in both prior surveys and the broader literature on antidepressant management [17,18,21,22]. Compared with other SNRIs, 41% of psychiatrists reported better adherence with duloxetine, while 40% found it similar. Although this proportion is lower than in our previous 80-psychiatrist survey (69%), the overall trend supports a clinician perception of duloxetine as at least equivalent or superior regarding adherence. Notably, the current study provides 95% confidence intervals for key outcomes (e.g., 41.0%, 95% CI: 31.9–51.2% for perceived better adherence), adding quantitative precision and highlighting variability in perceptions—an enhancement compared with prior surveys.

The most frequently endorsed strategies to improve adherence were detailed patient education (47%) and gradual titration starting at 30 mg/day (43%). Evidence suggests that gradual titration, starting at 30 mg/day, is associated with a significantly lower incidence of nausea and fewer early dropouts compared to starting directly at 60 mg/day (*p* = 0.003) [14,20]. This aligns with the clinical preference of most psychiatrists surveyed (78%), who reported initiating treatment at 30 mg/day followed by progressive titration based on response and tolerability. The majority of respondents (87%) also agreed that adherence would improve with more structured follow-up, including phone or digital reminders, reinforcing the clinical relevance of system-level support for treatment continuity.

### 4.4. Real-World Challenges in Adherence

Consistent with broader literature, recent clinical trials assessing interventions to improve adherence have shown mixed results. For instance, an open, multicenter study evaluating psychiatrist-focused education and patient/family collaborative care—including a reminder application—found that while both interventions significantly improved depression symptoms and mental quality of life, neither had a measurable effect on long-term antidepressant adherence [15]. Taken together, these results suggest the persistent difficulty in translating adherence-support strategies into sustained medication-taking behavior and underscore the relevance of understanding psychiatrists’ perceptions, which are likely to influence prescribing and monitoring practices. Furthermore, retrospective studies based on patient database records, such as Metrikin et al. (2025) with 9619 participants, indicate that while telephone and video visits may increase the likelihood of antidepressant prescribing, these prescriptions are less likely to be received and follow-up with the prescribing clinician is reduced compared with in-person visits [23]. This underscores the need for supportive healthcare system tools, such as mail-order pharmacies or electronic reminders, to ensure that virtual care decisions are effectively implemented [23].

### 4.5. Practice Setting, Demographics, and Novel Quantitative Insights

The present survey expands on prior work by providing exploratory subgroup analyses according to sex, age, practice setting, and professional experience. Male psychiatrists reported higher perceived efficacy than female psychiatrists (82.5%, 95% CI: 68.1–91.3% vs. 56.1%, 95% CI: 42.1–69.3%, χ^2^ = 6.24, *p* = 0.012). Stratified analyses also indicated variability across age and experience groups, although small cell sizes limited the ability to detect statistically significant differences in some subgroups. These quantitative analyses, confidence interval reporting, and subgroup exploration represent a novel contribution relative to our previous surveys, which primarily presented descriptive percentages without formal subgroup comparisons.

### 4.6. Comparison with Previous Surveys

Across all three surveys, psychiatrists consistently reported positive perceptions of duloxetine’s efficacy, tolerability, and role in adherence support. Despite variations in absolute percentages (likely due to differences in sample size, survey items, and population), the overall patterns remain congruent: duloxetine is viewed as effective for patients with MDD, GAD, and comorbid somatic or pain symptoms, is generally well tolerated, and is perceived to have at least equivalent adherence compared with other SNRIs or SSRIs. The consistency across surveys strengthens confidence in the robustness of clinician-reported perceptions while highlighting the importance of integrating subjective assessments with objective adherence measures in future studies [17,18].

The findings emphasize that successful psychopharmacological treatment depends not only on the intrinsic pharmacological properties of duloxetine but also on dosing strategy, patient education, and structured follow-up. Progressive titration, patient engagement, and proactive monitoring emerge as key strategies to optimize adherence and therapeutic outcomes, supported by both clinical trial evidence and real-world psychiatrist preferences. Additionally, the underutilization of digital tools reported by psychiatrists suggests an opportunity to integrate reminders, telepsychiatry, or collaborative care interventions to further enhance adherence, particularly in outpatient settings. Meta-analytic evidence further supports the use of multifaceted interventions within the Collaborative Care Model (CCM), which demonstrate the largest effects on adherence when patient, provider, and healthcare system factors are addressed simultaneously (OR 1.88, 95% CI: 1.40–2.54), with sustained but slightly attenuated effects at 12 months (OR 1.25, 95% CI: 1.02–1.53) [24].

Table 2 summarizes selected key survey outcomes with 95% confidence intervals, providing an integrative overview to support interpretation in the Discussion and to illustrate exploratory patterns across subgroups.

All values are descriptive and based on self-reported perceptions. Confidence intervals are approximate binomial estimates. Subgroup findings are exploratory and hypothesis-generating and should not be interpreted as confirmatory evidence.

### 4.7. Limitations

Several limitations should be noted. The survey relied on psychiatrists’ subjective perceptions rather than objective adherence measures such as pharmacy refill data or validated patient-reported outcomes, which may overestimate adherence due to recall or social desirability biases. The cross-sectional design precludes causal inference, and the sample size, although comparable with prior surveys, remains modest relative to the total population of Spanish psychiatrists. Small cell counts in certain subgroups limited statistical power, and direct head-to-head comparisons between antidepressants were not captured. Finally, generalizability may be restricted to Spanish healthcare contexts.

### 4.8. Future Directions

Future research should consider prospective registry studies combining clinician surveys with objective adherence data, comparative surveys across multiple SNRIs, and mixed-methods approaches incorporating patient interviews to triangulate adherence drivers. Interventions integrating structured follow-up and digital reminders, informed by the observed underuse of such tools, may be particularly valuable.

## 5. Conclusions

This national survey indicates that psychiatrists perceive duloxetine as an effective and well-tolerated option for major depressive disorder and generalized anxiety disorder, particularly in patients with comorbid somatic or pain symptoms. Adherence was perceived to benefit from lower starting doses, gradual titration, patient education, and structured follow-up. Novel aspects of this study include exploratory subgroup analyses and the reporting of 95% confidence intervals, which add quantitative precision and highlight professional variability beyond previous surveys. These findings should be interpreted cautiously given the subjective and exploratory nature of the data, but they support the clinical relevance of combining patient-centered prescribing strategies with structured follow-up to optimize treatment outcomes in routine practice.

## Data Availability

The data presented in this study are available on request from the corresponding author due to participants consented only to analyses conducted by the primary research team.

## Supplementary Materials

The following supporting information can be downloaded at: https://www.mdpi.com/article/10.3390/brainsci16020157/s1, Supplementary Materials S1: Survey on factors facilitating adherence to duloxetine treatment.

## Abbreviations

The following abbreviations are used in this manuscript:

MDD	Major depressive disorder
GAD	Generalized anxiety disorder
SNRI	Serotonin and norepinephrine reuptake inhibitor
