# Supplementary material for: Understanding Adherence to Duloxetine in Psychiatric Practice: A Cross-Sectional Evaluation of Clinicians’ Experience
Source: Brain Sci. 2026 Jan 29;16(2):157. doi: 10.3390/brainsci16020157 (PMC12938295; doi:10.3390/brainsci16020157)
Supplement: Supplementary file 1 [file brainsci-4065395-supplementary.pdf › brainsci-4065395-supplementary.pdf]

Supplementary Materials S1: Survey on factors facilitating adherence to duloxetine treatment

Survey on Factors Facilitating Adherence to Duloxetine Treatment

Dear Dr.,

Thank you for participating in this survey, which will take no more than 5 minutes to complete. Your responses will help us better understand the factors that influence adherence to duloxetine treatment in patients with depression and/or anxiety.

Demographic Data

1. Sex:

- Male
- Female
- Prefer not to answer

2. Age:

- 25–30 years
- 30–35 years
- 35–40 years
- 40–45 years
- 45–50 years
- 50–55 years
- 55–60 years
- 60–65 years

3. Province

4. Year of residency completion

5. Workplace:

- Outpatient clinic
- Acute inpatient unit
- Subacute inpatient unit
- Day hospital
- Therapeutic community

General Information

6. How often do you prescribe duloxetine to your patients with depression and/or anxiety?

A. Frequently (in more than 50% of cases)

B. Occasionally (in 20–50% of cases)

C. Rarely (in less than 20% of cases)

D. Never

Treatment Efficacy

7. In your experience, how would you rate the efficacy of duloxetine in the treatment of depression?

A. High

B. Moderate

C. Low

D. I do not consider it effective

8. How do you assess the efficacy of duloxetine in the treatment of anxiety?

A. Very effective

B. Quite effective

C. Slightly effective

D. Not effective

9. In which patient profile do you consider duloxetine to be most effective?

A. Patients with major depression without anxious symptoms

B. Patients with anxiety comorbid with depression

C. Patients with pain or other physical symptoms and depression

D. I do not observe significant differences among different profiles

Tolerability and Adverse Effects

10. Which adverse effect do you consider the most limiting for adherence to duloxetine treatment?

A. Nausea and gastrointestinal symptoms

B. Dizziness and fatigue

C. Sexual dysfunction

D. Increased blood pressure

11. What is the main reason why patients discontinue duloxetine treatment?
A. Perceived lack of efficacy

B. Intolerable adverse effects

C. Cost of the medication or limited access

D. Lack of education about the treatment (e.g., discontinuation when they feel better)

12. How often do your patients report adverse effects severe enough to lead to treatment discontinuation?

A. Frequently (in 20–50% of cases)

B. Occasionally (in 10–20% of cases)

C. Rarely (in less than 10% of cases)

D. Never or almost never

Dosing and Administration Factors

13. What do you consider to be the optimal initial dosing regimen to improve adherence?
A. 30 mg/day with subsequent titration

B. 60 mg/day from the start

C. 90 mg/day from the start

D. Depends on the patient

14. When do you observe better adherence to duloxetine treatment?
A. When the patient takes a single daily dose in the morning

B. When the patient takes a single daily dose at night

C. There is no difference in adherence depending on the time of administration

D. It depends on the individual tolerability of the patient

15. How long does it usually take for your patients to notice improvement with duloxetine?
A. Less than 2 weeks

B. Between 2 and 4 weeks

C. Between 4 and 8 weeks

D. More than 8 weeks

Patient- and Environment-Related Factors

16. Which factor do you believe has the greatest influence on your patients' adherence to duloxetine?

A. Family and social support

B. Education about the disease and treatment

C. Absence of significant adverse effects

D. Perceived treatment efficacy

17. How does adherence to duloxetine usually compare with other SNRIs (e.g., venlafaxine)?

A. Better adherence with duloxetine

B. Similar adherence to venlafaxine

C. Worse adherence with duloxetine

D. I do not observe significant differences

18. Which strategy do you think helps most to improve adherence to duloxetine?

A. Detailed education about adverse effects and benefits

B. Initiation with lower doses and gradual adjustment

C. Close follow-up during the first weeks

D. Use of digital tools (reminders, apps, etc.)

Psychiatrist's Perception of Adherence

19. In your experience, what percentage of patients discontinue duloxetine before 3 months of treatment?

A. Less than 20%

B. Between 20%–40%

C. Between 40%–60%

D. More than 60%

20. How often do you observe treatment discontinuation due to lack of therapeutic response?

A. Very frequently

B. Frequently

C. Infrequently

D. Rarely

21. What is patients' perception of duloxetine compared with other antidepressants?
A. More effective and better tolerated

B. Equally effective but with more adverse effects

C. Less effective than other antidepressants

D. I do not notice significant differences

Strategies to Improve Adherence

22. Do you usually recommend duloxetine in patients with a history of poor adherence to previous treatments?

A. Yes, with dosage adjustments

B. Depends on the patient profile

C. Only in selected cases

D. No, I prefer other options

23. Do you think adherence to duloxetine would improve with more structured follow-up (e.g., follow-up calls, more frequent visits, reminders)?
A. Yes, definitely

B. Probably yes

C. It would not make a significant difference

D. No, adherence depends more on patient motivation

24. How do you rate the overall satisfaction of patients who remain on duloxetine treatment for more than 6 months?

A. High satisfaction

B. Moderate satisfaction

C. Low satisfaction

D. I do not have enough information to assess this

25. Would you recommend duloxetine as a first-line treatment in patients with comorbid depression and anxiety?

A. Yes, in most cases

B. Only if other treatments have failed

C. Depends on the patient profile

D. No, I prefer other antidepressants as first-line options

Survey on factors facilitating adherence to duloxetine treatment (original Spanish version).

Encuesta sobre factores que facilitan adherencia en el tratamiento con duloxetina

Estimado/a Dr./Dra.,

Agradecemos su participación en esta encuesta, que no le ocupará más de 5 minutos. Sus respuestas nos ayudarán a entender mejor los factores que influyen en la adherencia al tratamiento con duloxetina en pacientes con depresión y/o ansiedad.

Datos demográficos

1. **Sexo:**

Hombre
Mujer
No contesta

2. **Edad**

25-30 años

30-35 años

35-40 años

40-45 años

45-50 años

50-55 años

55-60 años

60-65 años

3. **Provincia**

4. **Año de finalización de la residencia**

5. **Centro de trabajo**

Ambulatorio

Hospitalización de agudos

Hospitalización de subagudos

Hospital de Día

Comunidad terapéutica

Datos generales

6. **¿Con qué frecuencia prescribe duloxetina a sus pacientes con depresión y/o ansiedad?**

A. Frecuentemente (en más del 50% de los casos)

B. Ocasionalmente (entre el 20% y 50% de los casos)

C. Rara vez (menos del 20% de los casos)

D. Nunca

Eficacia del tratamiento

7. En su experiencia, ¿cómo valora la eficacia de duloxetina en el tratamiento de la depresión?

A. Alta

B. Moderada

C. Baja

D. No la considero eficaz

8. ¿Cómo evalúa la eficacia de duloxetina en el tratamiento de la ansiedad?

A. Muy eficaz

B. Bastante eficaz

C. Poco eficaz

D. No eficaz

9. ¿En qué perfil de pacientes considera que la duloxetina tiene mayor eficacia?

A. Pacientes con depresión mayor sin síntomas ansiosos

B. Pacientes con ansiedad comórbida a la depresión

C. Pacientes con dolor u otros síntomas físicos y depresión

D. No observo diferencias significativas en distintos perfiles

Tolerabilidad y y efectos adversos

10. ¿Cuál considera que es el efecto adverso más limitante para la adherencia al tratamiento con duloxetina?

A. Náuseas y síntomas gastrointestinales

B. Mareos y fatiga

C. Disfunción sexual

D. Aumento de la presión arterial

11. ¿Cuál es la principal razón por la que los pacientes interrumpen el tratamiento con duloxetina?

A. Falta de eficacia percibida

B. Efectos adversos intolerables

C. Coste del medicamento o acceso limitado

D. Falta de educación sobre el tratamiento (ejemplo: abandonan el tratamiento cuando sienten mejoría)

12. ¿Con qué frecuencia sus pacientes reportan efectos adversos de intensidad suficiente como para motivar el abandono del tratamiento?

A. Con frecuencia (en un 20-50% de los casos)

B. Ocasionalmente (en un 10-20% de los casos)

C. Raramente (menos del 10% de los casos)

D. Nunca o casi nunca

Factores posológicos y de administración

13. ¿Cuál considera que es el régimen de dosificación inicial óptimo para mejorar la adherencia?

A. 30 mg/día con titulación posterior

B. 60 mg/día desde el inicio

C. 90 mg/día desde el inicio

D. Depende del paciente

14. ¿Cuándo observa una mejor adherencia al tratamiento con duloxetina?

A. Cuando el paciente toma una única dosis diaria por la mañana

B. Cuando el paciente toma una única dosis diaria por la noche

C. No hay diferencia en la adherencia según la hora de administración

D. Depende de la tolerabilidad individual del paciente

15. ¿Cuánto tiempo suelen tardar sus pacientes en notar mejoría con duloxetina?

A. Menos de 2 semanas

B. Entre 2 y 4 semanas

C. Entre 4 y 8 semanas

D. Más de 8 semanas

Factores relacionados con el paciente y el entorno

16. ¿Qué factor cree que influye más en la adherencia de sus pacientes a duloxetina?

A. Apoyo familiar y social

B. Educación sobre la enfermedad y el tratamiento

C. Ausencia de efectos adversos significativos

D. Percepción de la eficacia del tratamiento

17. ¿Cómo suele ser la adherencia a duloxetina en comparación con otros IRSN (ej. venlafaxina)?

A. Mejor adherencia con duloxetina

B. Similar adherencia a venlafaxina

C. Peor adherencia con duloxetina

D. No observo diferencias significativas

18. ¿Qué estrategia cree que ayuda más a mejorar la adherencia a duloxetina?

A. Educación detallada sobre efectos adversos y beneficios

B. Inicio con dosis más bajas y ajuste progresivo

C. Seguimiento estrecho durante las primeras semanas

D. Uso de herramientas digitales (recordatorios, apps, etc.)

Percepción del psiquiatra sobre la adherencia

19. En su experiencia, ¿qué porcentaje de pacientes abandona duloxetina antes de los 3 meses de tratamiento?

A. Menos del 20%

B. Entre 20%-40%

C. Entre 40%-60%

D. Más del 60%

20. ¿Con qué frecuencia observa abandono del tratamiento debido a la falta de respuesta terapéutica?

A. Muy frecuente

B. Frecuente

C. Poco frecuente

D. Raro

21. ¿Cuál es la percepción de los pacientes sobre duloxetina en comparación con otros antidepresivos?

A. Más efectiva y mejor tolerada

B. Igual de efectiva pero con más efectos adversos

C. Menos efectiva que otros antidepresivos

D. No noto diferencias significativas

Estrategias para mejorar la adherencia

22. ¿Suele recomendar duloxetina en pacientes con historial de mala adherencia a tratamientos previos?

A. Sí, con ajustes en la dosificación

B. Depende del perfil del paciente

C. Solo en casos seleccionados

D. No, prefiero otras opciones

23. ¿Cree que la adherencia a duloxetina mejoraría con un seguimiento más estructurado (ej. llamadas de control, visitas más frecuentes, recordatorios)?

A. Sí, definitivamente

B. Probablemente sí

C. No haría una diferencia significativa

D. No, la adherencia depende más de la motivación del paciente

24. ¿Cómo valora la satisfacción general de los pacientes que siguen tratamiento con duloxetina durante más de 6 meses?

A. Alta satisfacción

B. Moderada satisfacción

C. Baja satisfacción

D. No tengo suficiente información para valorarlo

25. ¿Recomendaría duloxetina como fármaco de primera elección en pacientes con depresión y ansiedad comórbida?

A. Sí, en la mayoría de los casos

B. Solo si otros tratamientos han fallado

C. Depende del perfil del paciente

D. No, prefiero otros antidepresivos como primera opción
